# Amide Proton Transfer Contrast Distribution in Different Brain Regions in Young Healthy Subjects

**DOI:** 10.3389/fnins.2019.00520

**Published:** 2019-05-22

**Authors:** Thomas Sartoretti, Elisabeth Sartoretti, Michael Wyss, Árpád Schwenk, Arash Najafi, Christoph Binkert, Carolin Reischauer, Jinyuan Zhou, Shanshan Jiang, Anton S. Becker, Sabine Sartoretti-Schefer

**Affiliations:** ^1^Institute of Radiology, Cantonal Hospital Winterthur, Winterthur, Switzerland; ^2^Institute of Diagnostic and Interventional Radiology, University Hospital Zurich, University of Zurich, Zurich, Switzerland; ^3^Philips Health Systems, Zurich, Switzerland; ^4^Department of MR Research, Department of Radiology, Johns Hopkins University, Baltimore, MD, United States

**Keywords:** magnetic resonance imaging, amide proton transfer-weighted magnetic resonance imaging, molecular imaging, normal amide proton transfer-weighted signal intensity values, brain

## Abstract

**Objectives:**

To define normal signal intensity values of amide proton transfer-weighted (APTw) magnetic resonance (MR) imaging in different brain regions.

**Materials and Methods:**

Twenty healthy subjects (9 females, mean age 29 years, range 19 – 37 years) underwent MR imaging at 3 Tesla. 3D APTw (RF saturation B_1,rms_ = 2 μT, duration 2 s, 100% duty cycle) and 2D T2-weighted turbo spin echo (TSE) images were acquired. Postprocessing (image fusion, ROI measurements of APTw intensity values in 22 different brain regions) was performed and controlled by two independent neuroradiologists. Values were measured separately for each brain hemisphere. A subject was scanned both in prone and supine position to investigate differences between hemispheres. A mixed model on a 5% significance level was used to assess the effect of gender, brain region and side on APTw intensity values.

**Results:**

Mean APTw intensity values in the hippocampus and amygdala varied between 1.13 and 1.57%, in the deep subcortical nuclei (putamen, globus pallidus, head of caudate nucleus, thalamus, red nucleus, substantia nigra) between 0.73 and 1.84%, in the frontal, occipital and parietal cortex between 0.56 and 1.03%; in the insular cortex between 1.11 and 1.15%, in the temporal cortex between 1.22 and 1.37%, in the frontal, occipital and parietal white matter between 0.32 and 0.54% and in the temporal white matter between 0.83 and 0.89%. APTw intensity values were significantly impacted both by brain region (*p* < 0.001) and by side (*p* < 0.001), whereby overall values on the left side were higher than on the right side (1.13 vs. 0.9%). Gender did not significantly impact APTw intensity values (*p* = 0.24). APTw intensity values between the left and the right side were partially reversed after changing the position of one subject from supine to prone.

**Conclusion:**

We determined normal baseline APTw intensity values in different anatomical localizations in healthy subjects. APTw intensity values differed both between anatomical regions and between left and right brain hemisphere.

## Introduction

Amide proton transfer weighted (APTw) magnetic resonance (MR) imaging is a recently introduced contrast-agent free molecular imaging technique belonging to the chemical exchange saturation transfer (CEST) imaging (Van Zijl and Yadav, 2011; Wu et al., 2016; Van de Ven and Keupp, 2018). The signal in APTw imaging originates from amide protons in endogenous proteins and peptides in the brain parenchyma. Protons in amide groups exchange with water protons in their close vicinity. First amide protons are selectively saturated by a radiofrequency pulse tuned at a frequency of + 3.5 ppm from the water resonance. Second the saturation of the amide protons can be transferred to the adjacent water protons. Third if the saturation of the amide protons is continued for a longer time period (e.g., about 2 s), the resulting water saturation level is increased due to accumulation of multiple exchange events between amide and water protons. The resulting water saturation level, that is imaged by conventional MR technique, is strongly correlated with the concentration and exchange rate of proteins and peptides in the tissue (Van de Ven and Keupp, 2018).

In tumor tissue, especially in high grade malignant brain tumors, the content of mobile proteins and peptides is considerably elevated. This correlates with APTw intensity values and thus APTw imaging can be used for tumor characterization and diagnosis (Togao et al., 2014; Jiang et al., 2016, 2017a,b, 2019; Ma et al., 2016; Park et al., 2016; Su et al., 2017; Yu et al., 2017; Dreher et al., 2018, 2019; Van de Ven and Keupp, 2018; Zhou et al., 2019). Hence APTw imaging allows the differentiation of low-grade gliomas from high grade gliomas (Jiang et al., 2017a; Su et al., 2017; Van de Ven and Keupp, 2018), the prediction of the isocitrate dehydrogenase (IDH) mutation status in gliomas (Jiang et al., 2017b; Dreher et al., 2018), the differentiation of high grade gliomas from primary central nervous system lymphomas (Jiang et al., 2016) and from metastases (Yu et al., 2017) and the differentiation of tumor progression and treatment effects (Ma et al., 2016; Van de Ven and Keupp, 2018; Jiang et al., 2019). The technique has also been applied for the non-invasive detection of intracranial hemorrhage in various stages of blood degradation in acute, subacute and chronic stages (Ma et al., 2017) and of acute ischemic cerebral strokes (Park et al., 2016; Zhou et al., 2019).

To date no systematic analysis of APTw intensity values of different anatomical regions in normal brain parenchyma of young and healthy subjects has been performed. As there is growing interest in the application of APTw imaging in inflammatory, neurodegenerative and vascular diseases, as in Parkinson’s disease (Li et al., 2014, 2017) or multiple sclerosis frequently appearing in younger people, normal values of healthy brain parenchyma are of interest. Therefore we aimed at measuring normal APTw intensity values in different brain regions in young healthy subjects.

## Materials and Methods

### Study Subjects

The study was approved by the Cantonal Ethical Committee Zürich with BASEC Number 2018-01275. Written informed consent was obtained from all subjects in this study.

Twenty young healthy subjects, 11 males (mean age: 30 years, SD: 5 years, age range: 19–37) and 9 females (mean age: 28 years, SD: 4.5 years, age range: 20–37), with a mean age of 29 years and a range between 19 and 37 years were recruited for this study. There was no significant age difference between the gender groups (*p* = 0.5).

Inclusion criteria for young subjects was no T2 hyperintense foci in gray and white matter on 2D T2 turbo spin echo (TSE) images or any other abnormality as we aimed at defining normal APTw intensity values without any interference by clinically silent microangiopathy. Furthermore, subjects had to present without any current disease or history of disease and without cardiovascular risk factors (arterial hypertension, diabetes, hypercholesterolemia, smoking, coronary heart disease, cardiac arrhythmia, obesity, vasculitis). No subject had to be excluded.

### MR Imaging and Data Postprocessing

All subjects were scanned on a 3T scanner (Achieva, Philips Healthcare, Best, the Netherlands). An 8 channel head coil was used. The scanning parameters of the APT sequence (corresponding to the recently clinically approved APTw sequence of Philips Healthcare; Togao et al., 2017; Van de Ven and Keupp, 2018) are shown in Table 1. The APT sequence was scanned in transverse oblique orientation parallel to the intercommissural line. Ten slices with a slice thickness of 6 mm were acquired.

**Table 1:** Scan parameters of APTw sequence and of 2D T2w TSE sequence.

	3D APTw sequence	2D T2w TSE sequence
FOV	228 × 178 × 60 mm	230 × 230 × 165 mm
Acquisition voxel	1.8 × 1.8 × 6 mm	0.6 × 0.6 × 3.5 mm
Reconstruction voxel	0.9 × 0.9 × 6 mm	0.45 × 0.45 × 3.5 mm
Reconstruction matrix	256	512
Slice thickness	6 mm	3.5 mm
SENSE factor	1.6	1.5
Scan mode	3D spin echo	2D multislice spin echo
Fast imaging mode	Turbo spin echo multishot	Turbo spin echo multishot
TSE factor, profile order	174, linear	30, linear
Rest slabs	0	1
MultiVane percentage	–	160%
Flip angle (in degrees°)	90°	90°
TR and TE	TR 5,800–5,864 ms	TR 4,000 ms
	TE 7.8–8.3 ms	TE 120 ms
Fat suppression	SPIR	SPIR
APT	Saturation B_1rms:_ 2 μT Saturation duration: 2 s Continuos saturation, 100% duty cycle	No
Numbers of acquisitions NSA	1	1
Scan duration	03 min 42 s	03 min


To generate APTw imaging contrast, Magnetization Transfer Ratio asymmetry (MTR_asym_) was calculated with the following formula:

$$MTR_{asym}(\%)=\frac{\left(S_{-\Delta \omega }-S_{\Delta \omega }\right)}{S_{0}}$$

S_-Δω_ and S_Δω_ correspond to the water signal at negative and positive frequency offset, while S_0_ is the signal without radiofrequency saturation (Togao et al., 2017; Van de Ven and Keupp, 2018). In this study, nine image volumes at seven different frequency offsets (±3.1, ±3.5, ±3.9, and -1560 ppm) were acquired (Togao et al., 2017; Van de Ven and Keupp, 2018). A B_0_ map derived from three acquisitions at +3.5 ppm with slightly different echo shifts utilizing an mDIXON algorithm was used for a voxel-by-voxel B_0_ correction (Togao et al., 2017; Van de Ven and Keupp, 2018). B_1_ shimming was performed with each scan to allow for B_1_ inhomogeneity correction as described in detail by Togao et al. (2017). APTw intensity values in this paper always represent MTR_asym_ values at 3.5 ppm offset frequency (Δω), quantified by % water signal intensity (Togao et al., 2017; Van de Ven and Keupp, 2018).

For coregistration, a 2D T2w TSE sequence with 43 slices and 3.5 mm slice thickness was scanned; the sequence parameters of the two sequences are shown in Table 1.

**FIGURE 1:**
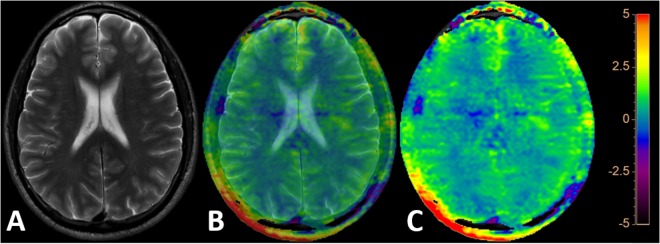
The pure 2D T2w TSE image **(A)**, a mixed 2D T2w TSE and APTw image (both each 50% contributing as shown in **B**) and the pure APTw color coded image **(C)** are depicted after fusion and coregistration of the 2D T2w TSE and the APTw image. “Rainbow” color coding is used depicting the APTw signal intensity from -5 to +5%.

Image postprocessing was obtained on an independent workstation “IntelliSpace Portal” version 8 (Philips Healthcare, Best, The Netherlands). APTw images were coregistered and overlayed with the acquired T2w TSE images. Coregistration was performed to ensure precise alignment of the two sequences if they were not fully geometrically identical. Color coding rainbow was used. After fusion and coregistration it was possible to switch from the pure T2w TSE image (Figure 1A), to the mixed T2w TSE and APTw image (both each 50% contributing as shown in Figure 1B) to the pure APTw color coded image (Figure 1C).

**FIGURE 2:**
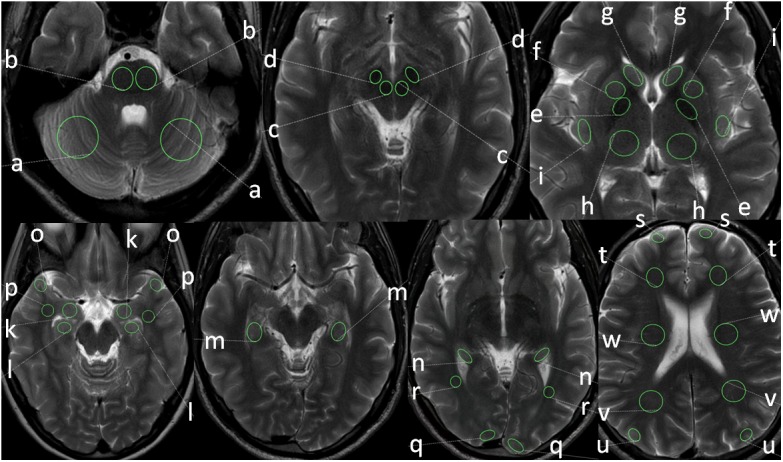
Positions and sizes of ROIs in different anatomical locations: cerebellar hemispheres (a), pons (b), red nucleus (c), substantia nigra (d), putamen (e), globus pallidus (f), head of caudate nucleus (g), thalamus (h), insular cortex (i), amygdala (k), head of hippocampus (l), body of hippocampus (m), tail of hippocampus (n), temporal cortex (o), temporal white matter (p), occipital cortex (q), occipital white matter (r), frontal cortex (s), frontal white matter (t), parietal cortex (u), parietal white matter (v), white matter of centrum semiovale (w).

Bilateral round or oval ROI measurements of APTw intensity values were performed in the following 22 regions by a senior neuroradiologist with 30 years of experience as demonstrated in Figure 2:

(a) Cerebellar hemisphere (a) and pons (b)
(b) Red nucleus (c) and substantia nigra (d)
(c) Putamen (e), globus pallidus (f), head of caudate nucleus (g), thalamus (h), insular cortex (i)
(d) Amygdala (k), head of hippocampus (l), body of hippocampus (m) and tail of hippocampus (n)
(e) Cortex (o) and white matter temporal lobe (p)
(f) Cortex (q) and white matter occipital lobe (r)
(g) Cortex (s) and white frontal lobe (t)
(h) Cortex (u) and white matter parietal lobe (v)
(i) White matter centrum semiovale (w)

Correct ROI positioning was controlled independently by a second neuroradiologist with 5 years of experience. The ROI size depended on the size of the anatomical structure and thus the size of the ROI was chosen based on the size of the anatomical region. Round and oval ROI sizes were used based on the anatomical configuration of the respective anatomical structure. The size of the ROI was smallest in the red nucleus (mean size 35 mm^2^) and largest in the centrum semiovale and cerebellum (410 mm^2^, each). ROI placement was done on the underlying T2w TSE image and the normal APTw intensity value was measured on the corresponding APTw image. In each subject APTw intensity values within the different ROIs in each anatomical region were obtained for mean APTw as well as for minimum APTw and maximum APTw (as the minimum and maximum values are of potential clinical interest; Yu et al., 2017) with the left and the right hemisphere separately and the corresponding values were calculated for both hemispheric sides combined (bihemispheric).

Ultimately a male subject was scanned once in prone and in supine position to further investigate left-right differences in brain regions.

### Statistical Analysis

APTw values were reported with mean, standard deviation and the mean minimum and maximum values for each side and brain region. The data was analyzed with a mixed model to assess the impact of side, gender and brain region on mean APTw values. The model consisted of a random intercept and random slope for side and the fixed effects brain region, side, and gender as well as the interaction of brain region and side. The anova function for mixed models was applied (Wald statistics). To assess possible age differences between genders a two-sided student’s *t*-test was used.

The analysis was performed in the R programming language (version 3.3.3, R Core Team, 2017). The complete statistical report is provided as Supplementary Information.

## Results

**FIGURE 3:**
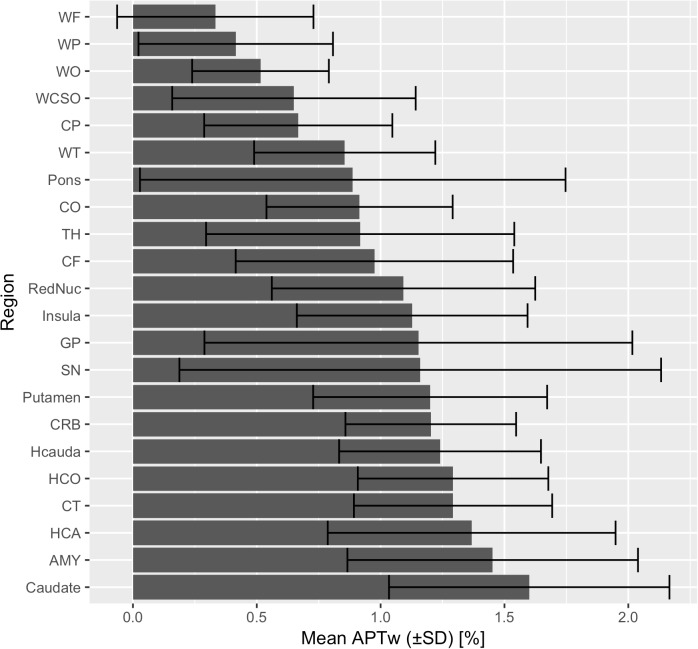
Mean APTw intensity values (bihemispheric) with standard deviations are shown for each brain region in ascending order.

In all subjects there were no apparent motion artifacts. The results of the averaged APTw mean, standard deviation, minimum and maximum intensity values in different brain regions, separate for various anatomical structures, are presented in Table 2, in the bar chart in Figure 3 as well as in the online supplement. The APTw intensity values are separately shown for the left and the right hemisphere and combined for both sides (bihemispheric).

**Table 2:** Normal APTw intensity values.

Mean APTw intensity values ± standard deviation plus minimum and maximum APTw intensity values in brackets	APTw intensity values left hemisphere	APTw intensity values right hemisphere	APTw intensity values bihemispheric
Cerebellum	1.29 ± 0.35; [0.83, 1.85]	1.12 ± 0.32; [0.61, 1.64]	1.2 ± 0.34; [0.72, 1.74]
Pons	0.92 ± 0.94; [-0.79, 2.98]	0.86 ± 0.80; [-0.96, 2.77]	0.89 ± 0.86; [-0.87, 2.87]
Red Nucleus	1.26 ± 0.58; [0.33, 2.3]	0.93 ± 0.44; [-0.06, 1.97]	1.09 ± 0.53; [0.14, 2.13]
Substantia nigra	1.58 ± 0.79; [0.22, 3.32]	0.75 ± 0.97; [-0.62, 2.33]	1.16 ± 0.97; [-0.2, 2.83]
Head of caudate nucleus	1.84 ± 0.54; [0.79, 2.73]	1.37 ± 0.50; [0.16, 2.3]	1.6 ± 0.57; [0.47, 2.52]
Globus pallidus	1.54 ± 0.79; [0.53, 2.55]	0.77 ± 0.77; [-0.2, 1.65]	1.15 ± 0.86; [0.17, 2.1]
Putamen	1.37 ± 0.45; [0.7, 2.05]	1.03 ± 0.44; [0.33, 1.71]	1.2 ± 0.47; [0.52, 1.88]
Thalamus	1.11 ± 0.47; [-0.13, 2.31]	0.73 ± 0.70; [-0.46, 1.89]	0.92 ± 0.62; [-0.29, 2.1]
Insula	1.11 ± 0.45; [0.47, 1.74]	1.15 ± 0.50; [0.49, 1.78]	1.13 ± 0.47; [0.48, 1.76]
Amygdala	1.57 ± 0.67; [0.56, 2.76]	1.34 ± 0.48; [0.51, 2.1]	1.45 ± 0.59; [0.53, 2.43]
Hippocampal head	1.47 ± 0.66; [0.53, 2.34]	1.27 ± 0.49; [0.43, 2.13]	1.37 ± 0.58; [0.48, 2.23]
Hippocampal body	1.39 ± 0.47; [0.47, 2.32]	1.2 ± 0.26; [0.5, 1.97]	1.29 ± 0.38; [0.48, 2.14]
Hippocampal tail	1.35 ± 0.40; [0.59, 2.05]	1.13 ± 0.39; [0.56, 1.73]	1.24 ± 0.41; [0.57, 1.89]
Cortex frontal	0.92 ± 0.62; [-0.23, 2.17]	1.03 ± 0.50; [-0.14, 2.38]	0.98 ± 0.56; [-0.18, 2.27]
Cortex occipital	1.02 ± 0.34; [0.49, 1.64]	0.81 ± 0.39; [0.27, 1.75]	0.92 ± 0.38; [0.38, 1.69]
Cortex temporal	1.37 ± 0.43; [0.63, 1.97]	1.22 ± 0.37; [0.6, 1.87]	1.29 ± 0.4; [0.61, 1.92]
Cortex parietal	0.78 ± 0.35; [0.15, 1.47]	0.56 ± 0.38; [-0.06, 1.15]	0.67 ± 0.38; [0.05, 1.31]
White matter frontal	0.35 ± 0.45; [-0.09, 0.78]	0.32 ± 0.35; [-0.12, 0.8]	0.33 ± 0.4; [-0.1, 0.79]
White matter occipital	0.54 ± 0.23; [0.26, 0.81]	0.5 ± 0.32; [0.24, 0.76]	0.52 ± 0.28; [0.25, 0.78]
White matter temporal	0.89 ± 0.34; [0.33, 1.49]	0.83 ± 0.40; [0.36, 1.38]	0.86 ± 0.37; [0.34, 1.43]
White matter parietal	0.45 ± 0.40; [-0.03, 0.94]	0.39 ± 0.40; [-0.19, 1.64]	0.42 ± 0.39; [-0.11, 0.9]
White matter centrum semiovale	0.82 ± 0.48; [-0.19, 1.64]	0.48 ± 0.45; [-0.57, 1.52]	0.65 ± 0.49; [-0.38, 1.58]


Mean APTw intensity values in the hippocampus and amygdala varied between 1.13 and 1.57%, in the deep subcortical nuclei (putamen, globus pallidus, head of caudate nucleus, thalamus, red nucleus, substantia nigra) between 0.73 and 1.84%, in the frontal, occipital and parietal cortex between 0.56 and 1.03%; in the insular cortex between 1.11 and 1.15%, in the temporal cortex between 1.22 and 1.37%, in the frontal, occipital and parietal white matter between 0.32 and 0.54% and in the temporal white matter between 0.83 and 0.89%.

APTw intensity values of female and male subjects were pooled as gender was no significant predictor (*p* = 0.240) within the mixed model.

**FIGURE 4:**
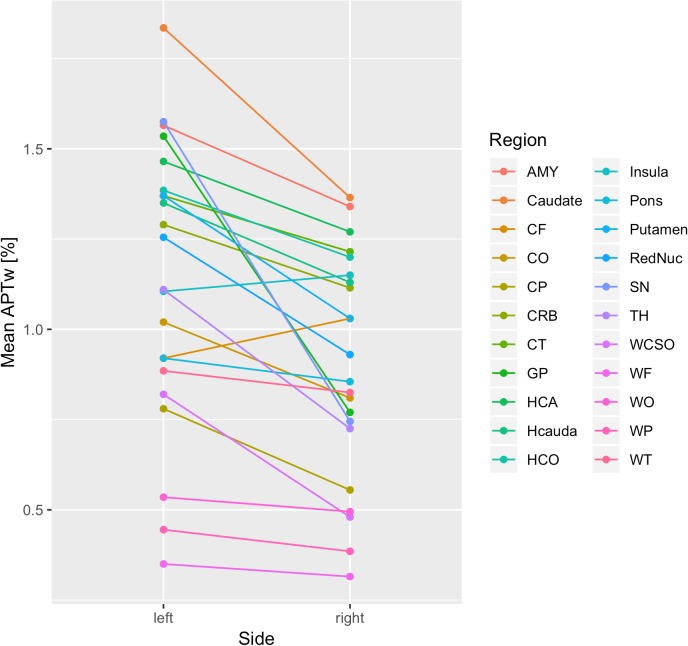
Differences in Mean APTw intensity values between sides are visualized.

**FIGURE 5:**
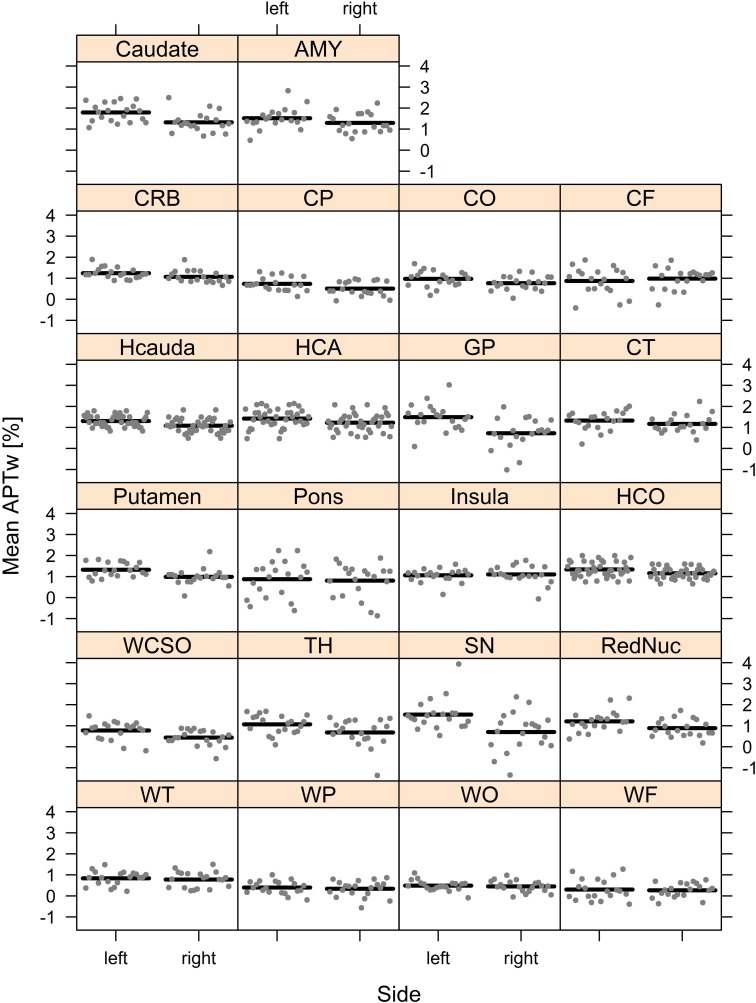
Mean APTw intensity values for all brain regions (sides separated) are visualized.

**FIGURE 6:**
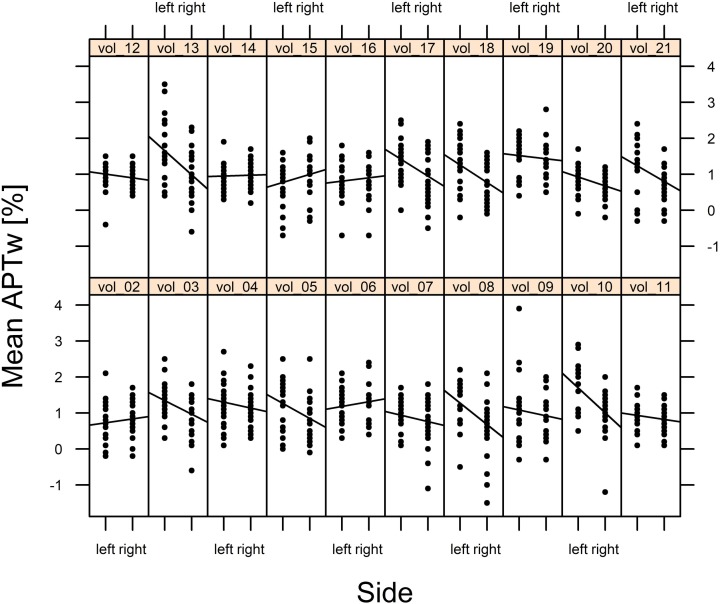
Mean APTw intensity values of every brain region of each subject (sides separated) are visualized.

However, the Wald statistics of the model suggested that mean APTw values were significantly impacted by side (*p* < 0.001), brain region (*p* < 0.001), and the interaction between side and brain region (*p* < 0.001). Thus, the effect of side essentially depended on the brain region. While for most brain regions, mean APTw intensity values were higher for the left than the right side, the frontal cortex and the insula presented with slightly higher values on the right side. Nonetheless the significant impact of side on mean APTw intensity values was reflected in the difference between overall left and right mean APTw values when considering all brain regions (1.13% on the left side vs. 0.9% on the right side, difference: 0.23%). Furthermore, differences between values for the left and right side were highest in the substantia nigra (difference: 0.83%) and globus pallidus (difference: 0.77%) and tend toward zero for white matter temporal lobe (difference: 0.06%), white matter parietal lobe (difference: 0.06%), white matter occipital lobe (difference: 0.04%), white matter frontal lobe (difference: 0.04%) and the pons (difference: 0.07%). These differences are visualized in Figure 4, 5 and are shown quantitatively in Table 2. Ultimately the side difference in mean APTw values was also recognizable when examining the data of single subjects. Of 20 subjects, 15 presented with higher values on the left side when considering all brain regions, while 5 subjects present with slightly higher values on the right side as seen in Figure 6.

To investigate these findings we examined one male subject in prone position after the regular scan in supine position. The differences in APTw intensity values between the left and the right side were partially reversed after changing the position of the subject from supine to prone. The distribution of APTw intensity values between brain regions did not change. We decided not to substantiate these findings with more subjects, since the prone position was very poorly tolerated.

## Discussion

In the present study, we report normal APTw intensity values in healthy, young subjects. The averaged APTw mean, minimum and maximum intensity values were calculated after ROI measurements in 22 different brain regions. It is important to know the normal APTw intensity values in different brain regions before pathologic conditions can be studied and analyzed with this new technique. Moreover, we performed ROI measurements for the left and the right hemisphere and found that the side significantly impacted APTw intensity values.

In general, cortical areas always presented with higher mean APTw intensity values than white matter, but also differences in APTw intensity values between different cortical areas and subcortical nuclei were obvious. APTw intensity values in the frontal, occipital and parietal cortex were very similar with mean APTw intensity values between 0.55 and 1.08%. These mean APTw intensity values were the lowest cortical values measured followed by higher mean APTw intensity values in the insular cortex and the highest mean APTw intensity values in the hippocampus and the temporal cortex.

Previously, normal APTw intensity values have only been described for 5 brain regions in older, healthy individuals (age range ∼54–77 years) without analyzing differences between the left and right brain hemisphere as measurements from both sides were pooled (Li et al., 2014, 2017). The study was performed on a 3T MR scanner with a saturation duration of 200 ms × 4 and a power level of 2 uT. We report slightly lower mean values in the substantia nigra, the red nucleus and considerably higher values in the putamen, globus pallidus and the head of the caudate (Li et al., 2014, 2017). Furthermore, APTw intensity values of brain regions of patients with early and advanced stage Parkinson’s disease have been reported. The pathologic values described seem to partially overlap with the values described in this study. For example, in the substantia nigra in patients with early stage Parkinson’s disease a mean APTw intensity value of 1.1% was described, while a value of 1.3% was reported for the corresponding control group of healthy elderly individuals (Li et al., 2014). We report a mean value of 1.16% as a value encountered in healthy young individuals. Thus all three values are close together which further emphasizes the need to first establish normal values in healthy subjects (Li et al., 2014, 2017). Possibly the difference in values between the control group (1.3%) and the value reported in this study (1.16%) are caused by the age difference in the subjects examined. However, the impact of age on APTw intensity values is unknown and should be investigated in further studies. Additionally, in the same study, for example, the difference in values between the globus pallidus of healthy brain regions and the corresponding region of patients with advanced stage Parkinson’s disease is reported as 0.3% (Li et al., 2014, 2017). In this study we found that overall values on the left side were on average 0.23% higher than on the right side. Within the globus pallidus, we reported the left side to be on average 0.77% higher than on the right side. Thus these side differences might have impacted the results described above (Li et al., 2014, 2017). This indicates that caution has to be applied when using APTw imaging in a quantitative manner and that previous studies using APTw intensity values quantitatively should be judged accordingly. Ultimately, numerous studies utilizing various APTw imaging sequences have been published (Li et al., 2014, 2017; Togao et al., 2014; Jiang et al., 2016; Ma et al., 2016; Park et al., 2016; Dreher et al., 2018, 2019). A comparison between these studies may be difficult, as the sequence parameters of the APTw sequences may impact the results. In this study we used the clinically approved APTw sequence by Philips Healthcare (Togao et al., 2017; Van de Ven and Keupp, 2018). As this is the only clinically approved APTw imaging sequence currently commercially available, results from future clinical studies should be comparable to the results obtained in this study.

In this study we found large differences in mean APTw values between cortical areas, yet how can these be explained? Differences in the histological structure and corresponding regional metabolite content of the insular cortex and the hippocampal cortex compared to the lobar cortex may be responsible for our findings. The insular cortex for example has three special areas that differ in cytoarchitecture: the granular (classic six-layered structure), dysgranular (with only thin layer 4) and agranular subdivision (with lack of layer 4). These three subdivisions are strongly interconnected (Nieuwenhuis, 2012; Gogolla, 2017). The neurons of the cerebral lobar cortex, on the other hand, are classically divided into six main layers. The differences in lamination of the cerebral cortex help to classify the cortex into the neocortex with 6 layers as seen in the frontal, parietal, temporal, and occipital cortices and the allocortex with 3–4 layers as seen in the hippocampus and the insula (Nieuwenhuis, 1994; Fatterpekar et al., 2002).

In MR spectroscopy, differences in the metabolite concentration in different brain regions are reported as well. High choline peaks are demonstrated in insular cortex, hippocampus, in the thalamus, in the cerebellum and in the pons. High levels of creatine are reported in the cerebellum and low levels in pons and parieto-occipital gray and white matter. NAA has the highest level in pons and low levels in the allocortex. These regional metabolite variations reflect the different histology of different brain regions and are thus possibly reflected in differing APTw intensity values as well (Jacobs et al., 2001; Arslanoglu et al., 2004).

Additionally, the iron content in caudate nucleus, putamen, globus pallidus, substantia nigra, and red nucleus, bound to ferritin protein, may have an influence on the higher APTw intensity value in these subcortical nuclei (Aquino et al., 2009). Susceptibility interference at the skull base may also cause higher APTw intensity values in the temporal lobe both in the cortex and in the white matter.

Overall we observed higher APTw intensity values in gray matter regions than in white matter regions, as described in two previous studies (Jin et al., 2013; Xu et al., 2016). Both studies have attributed the higher APTw intensity values in gray matter regions to higher concentrations of mobile proteins and peptides in gray matter than in white matter. Furthermore, the differences of values between left and right hemisphere described in this study are already known for apparent diffusion coefficient ADC (Naganawa et al., 2003) and for volume of gray matter in different brain regions (Watkins et al., 2001). It has been speculated that the volumetric differences are due to real hemispheric differences related to functional hemispheric specialization correlated with structural differences (Watkins et al., 2001). It is known that two similar hemispheric areas on the left and the right side have a differing number of neurons and interhemispheric connections and that the amount on non-neuronal space varies (Watkins et al., 2001). The thickness of the myelin sheath differs between the left and the right side as well and an increased thickness of the myelin sheath is reported for the left temporal lobe (Watkins et al., 2001). Interestingly, hemispheric differences of APTw intensity values have been previously described in grade IV gliomas at 7T (Dreher et al., 2019), yet no hemispheric differences were observed in their healthy control group.

Besides anatomical reasons, the hemispheric differences in APTw intensity values may also be affected by a variety of technical factors (Sun et al., 2007; Zhang et al., 2007, 2017; Van Zijl and Yadav, 2011; Wu et al., 2016; Heo et al., 2017; By et al., 2018; Van de Ven and Keupp, 2018; Zu, 2018; Zhou et al., 2019). Water longitudinal relaxation time (T_1_) has been shown to influence the APTw signal. There are two types of T_1_ effect, namely T_1_ recovery and T_1_-related saturation. Depending on the level of direct water saturation effects, the influence of T_1_ effects on the APTw signal is either linear or complex. This relationship further depends on the field strengths of the MR scanner, irradiation power and whether a non-steady-state acquisition or steady-state acquisition is performed. Specifically an irradiation power of 2 μT (as applied in our APTw sequence) is recommended for robust clinical APTw imaging at 3T (Heo et al., 2017; Zhang et al., 2017). At 2 μT in clinical MRI systems (in comparison to high field MRI systems) the APTw signal can be considered roughly insensitive to T_1_ effects, even though slight residual effects may still remain (Zu, 2018). Furthermore it is known that semi-solid magnetization transfer (MT) and other nearby CEST and relayed nuclear Overhauser enhancement (rNOE) saturation transfer effects can impact the APTw signal (Zhang et al., 2017).

Ultimately B_1_ effects stemming from a slightly imperfect distribution of the 2 μT irradiation power across the brain may also impact APTw intensity values (Zhao et al., 2011; By et al., 2018).

Different technical approaches are used and applied for correction of the B_0_ and B_1_ field effects in CEST imaging with the commercially available APTw sequence used in this study included (Sun et al., 2007; Zhou et al., 2013; Manoliu et al., 2015; Windschuh et al., 2015; Khlebnikov et al., 2017; Togao et al., 2017; Van de Ven and Keupp, 2018). Possible residual B_1_ effects may, however, explain why hemispheric differences in APTw intensity values of some brain regions were reversed after changing the position of a subject from supine to prone while the distribution of APTw intensity values between brain regions remained the same. However, in the previous study that reported hemispheric differences in grade IV gliomas (Dreher et al., 2019), B_1_ effects could be ruled out and thus the reversed values in our subject may simply represent a coincidence.

We did not further investigate these findings as firstly, the prone position was very poorly tolerated and secondly, a detailed investigation of these findings is beyond the scope of this paper as we primarily aimed at defining the normal APTw contrast distribution in different brain regions. Nonetheless we believe that further investigations should be conducted to determine whether these hemispheric differences stem from anatomical differences or possibly technical factors.

To sum up, a combination of all of these factors together with anatomical differences may explain the hemispheric differences of APTw intensity values observed in our study and may also explain the contrast distribution between different brain regions. Based on our findings we recommend that the averaged APTw intensity value, i.e., the mean of the left and the right hemispheric value, may be considered as the normal value. Finally, when APTw imaging is used qualitatively in brain tumor analysis, these slight differences in signal intensity values cannot be distinguished on the color-coded maps by visual inspection. For tumor grading it is recommended to compare the APTw intensity values with healthy contralateral brain parenchyma (Van de Ven and Keupp, 2018). We suspect that knowledge of hemispheric differences of the APTw signal potentially influenced by technical factors, could even further improve reliability of tumor grading with APTw imaging. When APTw intensity values are used, however, these slight differences should be taken into account (Li et al., 2014, 2017).

There are several limitations which must be acknowledged: First the sample size is fairly small, though in the typical range for pilot studies establishing baselines in healthy subjects. Nonetheless, dependency of physiological APTw intensity values on gender, age and shape of the head and body weight should be assessed in more detail in future studies.

ROI measurements are a common and well-established method to quantitatively assess APTw tissue values (Li et al., 2014, 2017; Togao et al., 2015). Furthermore, a high degree of scan-rescan reproducibility and repeatability of values calculated with ROI measurements has been demonstrated for APTw imaging (Li et al., 2014, 2017; Togao et al., 2015; Khlebnikov et al., 2017). Nonetheless, pixel-wise segmentation of brain regions (for example of the hippocampus and the subcortical nuclei) and secondary APTw intensity value measurement of the segmented brain structures might give better APTw intensity normal values than ROI measurements. However, even if fully segmented, some structures very close to the ventricular system (i.e., red nucleus, head of caudate nucleus, hippocampus, amygdala, parietal white matter) and thus to CSF will still be affected by CSF flow or pulsation artifacts. Additionally, slice thickness was different between T2w TSE images and APTw images leading to possible partial volume effects. Therefore, CSF flow or pulsation artifacts as well as partial volume effects may have influenced the measurements of the APTw intensity values within the ROIs (a problem known from diffusion weighted imaging; Schmidt et al., 2016; Surer et al., 2018). We did not investigate intra- or interreader reproducibility in our subjects because this has already been examined by other groups (Togao et al., 2015; Schmidt et al., 2016; Van de Ven and Keupp, 2018).

Further studies are necessary to thoroughly analyse the effect of various technical factors on the hemispheric differences in APTw intensity values with dedicated phantom model experiments and subject experiments.

Lastly, due to the single-center design of our study, measurements were only performed on data obtained from a single scanner. We believe, that further studies on different models of MR scanners are necessary to confirm our data.

## Conclusion

We determined normal APTw intensity values in 22 different brain regions in healthy, young subjects. APTw intensity values differed considerably between different anatomical structures, possibly related to histological differences of the various brain tissues, susceptibility interference, flow and partial volume effects. Hemispheric differences in APTw intensity values in this study might be explained by anatomical differences related to hemispheric specialization and secondary structural differences and possibly by a variety of effects influencing the APTw signal. Therefore, we recommend using the bihemispheric, averaged APTw intensity value as a normal value.

## Data Availability

A full statistical report and all data is available as Supplementary Information.

## Ethics Statement

This study was carried out in accordance with the recommendations of the Cantonal Ethical Committee Zürich with written informed consent from all subjects. All subjects gave written informed consent in accordance with the Declaration of Helsinki. The protocol was approved by the Cantonal Ethical Committee Zürich.

## Author Contributions

TS, ES, AB, SS-S, MW, JZ, and SJ designed the study and interpreted the results. TS, SS-S, MW, ÁS, and AN performed the experiments. AB, JZ, MW, TS, and ES analyzed the data. TS, SS-S, AB, and MW wrote the manuscript. CB and CR provided technical advice. All co-authors contributed constructively to the manuscript.

## Conflict of Interest Statement

MW was a part time employee of Philips Healthcare Switzerland. JZ was a co-inventor on a patent for the APT MRI technology. This patent is owned and managed by Johns Hopkins University. The remaining authors declare that the research was conducted in the absence of any commercial or financial relationships that could be construed as a potential conflict of interest.

## Abbreviations

AMY: amygdale
APT: amide proton transfer
Caudate: head of caudate nucleus
CF: frontal cortex
CO: occipital cortex
CP: parietal cortex
CRB: cerebellum
CT: temporal cortex
GP: globus pallidus
HCA: hippocampal caput (head)
HCauda: hippocampal cauda (tail)
HCO: hippocampal corpus (body)
li: left side
MT: magnetization transfer
MTRasym: Magnetization Transfer Ratio asymmetry
re: right side
Red Nuc: red nucleus
rNOE: relayed nuclear Overhauser enhancement
S-Δω: signal at negative frequency offset -Δω
SΔω: signal at positive frequency offset Δω
S0: signal without radiofrequency saturation
SN: substantia nigra
T2w: T2 weighted
TFE: turbo field echo
TH: thalamus
TSE: turbo spin echo
WCSO: white matter centrum semiovale
WF: frontal white matter
WO: occipital white matter
WP: parietal white matter
WT: temporal white matter
