# Supplementary material for: Amide Proton Transfer Contrast Distribution in Different Brain Regions in Young Healthy Subjects
Source: Front Neurosci. 2019 May 22;13:520. doi: 10.3389/fnins.2019.00520 (PMC6538817; doi:10.3389/fnins.2019.00520)
Supplement: Supplementary file 1 [file Data_Sheet_1.pdf]

APTw Imaging of the brain

graf biostatistics • Amelenweg 5 • 8400 Winterthur
phone 052 202 83 14 • mobile 079 270 67 72
graf@biostatistics.ch • www.biostatistics.ch

Statistical Report

Nicole Graf (graf@biostatistics.ch)

Version 1.0 of 09/01/2019

- Data
- Statistical analysis
- Software
- Results
  - Data visualisation
  - Mixed model
  - Assumptions
    - Assessing assumptions on the within-group error
    - Assessing assumptions of the random effect
- Packages
- References

Data

This analysis is based on the data file “APT Reference Values_Graf.xlsx” received on January 3, 2019, via e-mail.

Statistical analysis

The data was analysed with a mixed model. The final model consisted of a random intercept and random slope for side and the fixed effects brain area, side, and gender as well as the interaction of brain area and side. The assumptions on the within-group error were checked with scatter plots of the standardized residuals versus fitted values and a normal plot of the residuals. The assumptions of the random effect was checked with a normal plot of the estimated random effects.

Software

All analyses were performed in the R programming language (version 3.3.3) (R Core Team, 2017). The package “nlme” (Pinheiro J, Bates D, DebRoy S, Sarkar D, and R Core Team, 2017) was used to compute the mixed model. The package “visreg” (Breheny P and Burchett W, 2017) was used to visualise the fitted model.

Results

Data visualisation

Figure 1 shows APTw imaging values of all brain areas and patients for left and right side.

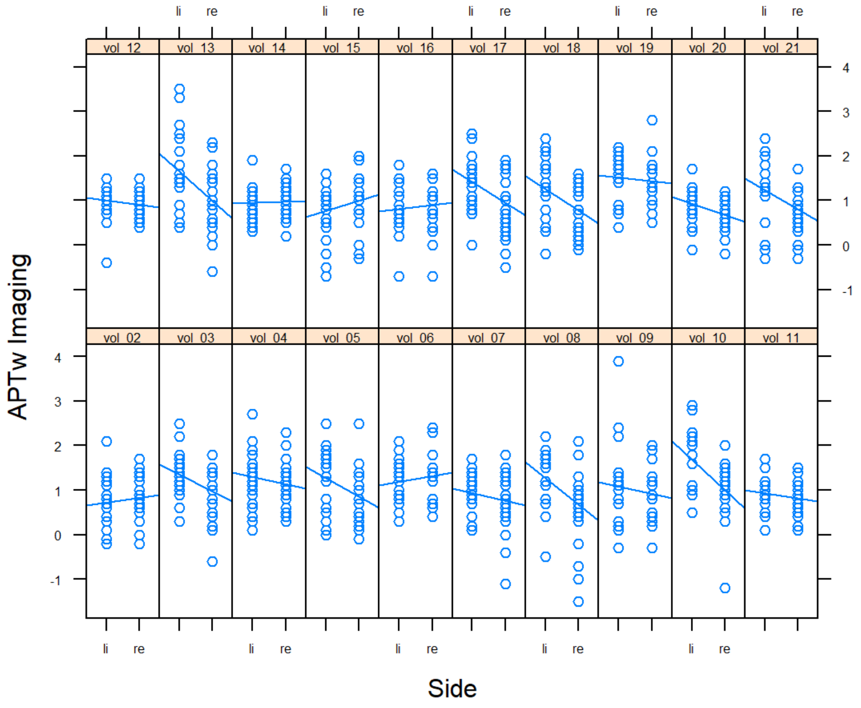

Figure 1. APTw imaging values versus side for all patients.

Mixed model

The interaction between brain area and side proved to be significant and was therefore included in the model. The comparison between a random intercept model (“model1”) and a random intercept and slope model (“model2”) indicated that the random intercept and slope model performed better. The anova output of “model2” suggests significant effects for side (p<0.001), area of the brain (p<0.001), and the interaction between side and area of the brain (p<0.001). Gender was no significant predictor (p=0.240). Thus, the effect of side essentially depends on the brain area. Figure 2 visualises the fitted model (gender is set to its most common category, i.e., male). Figure 2 illustrates that for most brain areas values for APTw imaging are higher for the left than the right side, except for “CF” and “Insula”, where values are slightly higher for the right than the left side. Differences (“diff”) between predicted values for left and right side vary between the brain areas, thus, they are highest for “SN” and “GP” and tend towards zero for “WT”, “WP”, “WO”, “WF”, and “Pons” (see also last table of differences between predicted values for left and right side).

```
##        Model df      AIC      BIC    logLik   Test  L.Ratio p-value
## model1     1 47 1589.182 1817.682 -747.5910
## model2     2 49 1555.435 1793.659 -728.7176 1 vs 2 37.74688  <.0001
```

```
##                 numDF denDF  F-value p-value
## (Intercept)         1   937 589.3745  <.0001
## Seite               1   937  14.0034  0.0002
## Hirnareal          21   937  22.8618  <.0001
## gender              1    18   1.4793  0.2396
## Seite:Hirnareal    21   937   2.4711  0.0003
```

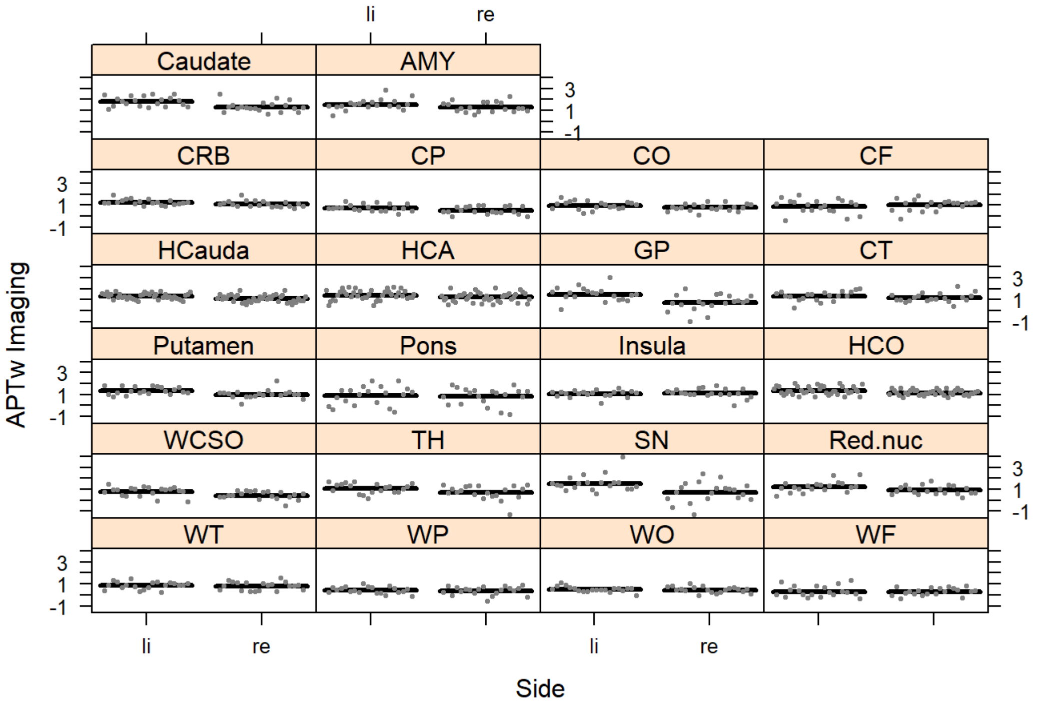

Figure 2. The estimated relationship between ATPw imaging and side for all brain areas. Gender is set to its most common category, i.e., male.

```
##    Hirnareal   diff
## 83        CF -0.110
## 65    Insula -0.045
## 51        WF  0.035
## 49        WO  0.040
## 45        WT  0.060
## 47        WP  0.060
## 63      Pons  0.065
## 75        CT  0.155
## 77       CRB  0.175
## 67       HCO  0.185
## 71       HCA  0.195
## 81        CO  0.210
## 69    HCauda  0.220
## 79        CP  0.225
## 87       AMY  0.225
## 59   Red.nuc  0.325
## 61   Putamen  0.340
## 53      WCSO  0.340
## 55        TH  0.385
## 85   Caudate  0.470
## 73        GP  0.765
## 57        SN  0.830
```

Assumptions

Assessing assumptions on the within-group error

The residuals are centered at 0, and the variability seems to be constant (Fig. 3). The residuals are slightly s-shaped, thus, a minor deviation from normality (Fig. 4).

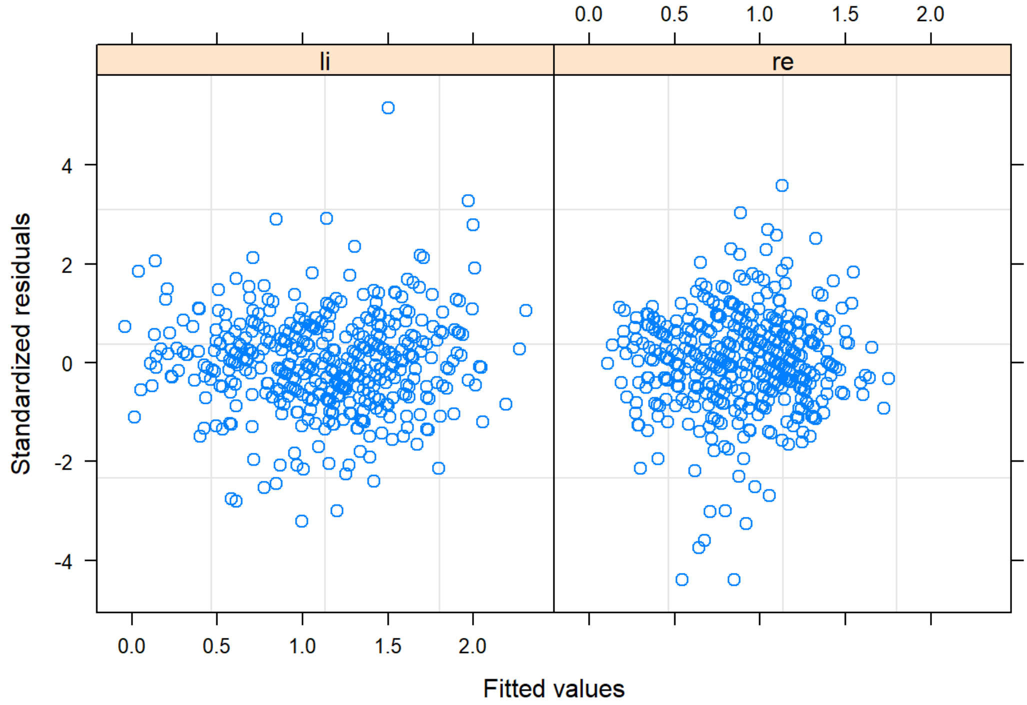

Figure 3. Scatter plots of standardized residuals versus fitted values for the heteroscedastic fit of model2 by side.

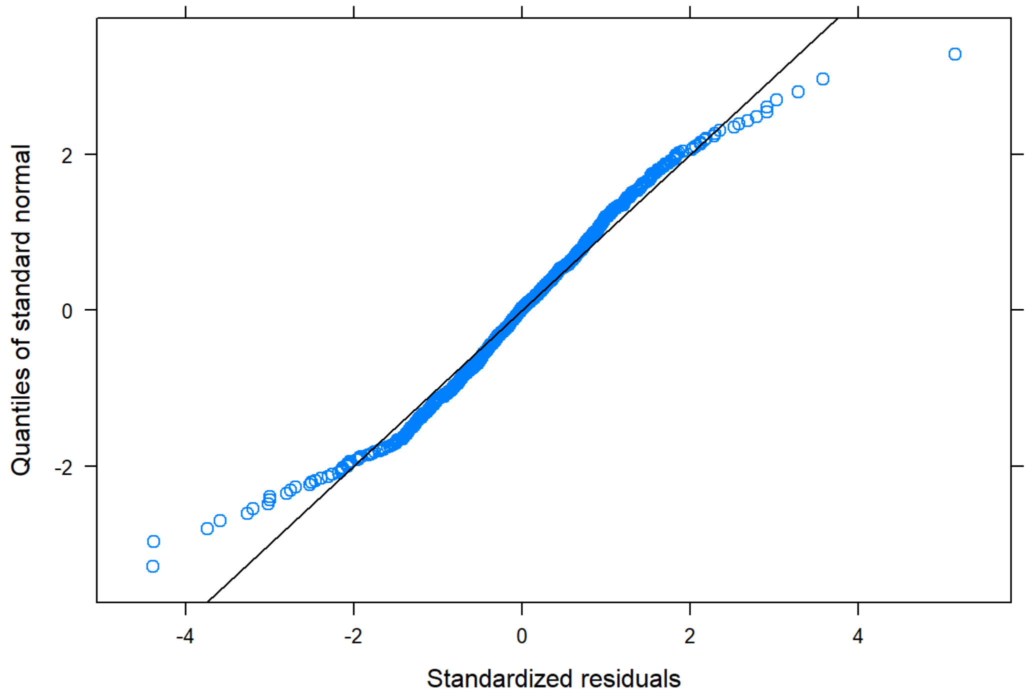

Figure 4. Normal plot of residuals for the model2 fit.

Assessing assumptions of the random effect

The assumption of normality of random effect seems reasonable.

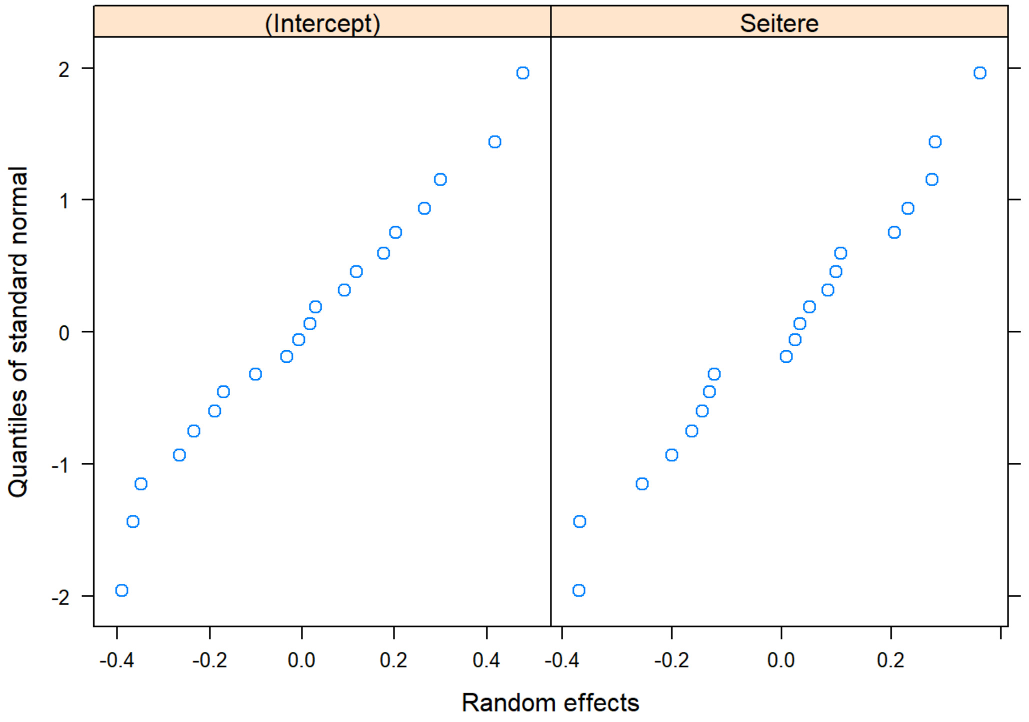

Figure 6. Normal plot of estimated random effects for the model2 fit.

Packages

```
## R version 3.3.3 (2017-03-06)
## Platform: x86_64-w64-mingw32/x64 (64-bit)
## Running under: Windows 10 x64 (build 17134)
##
## locale:
## [1] LC_COLLATE=German_Switzerland.1252  LC_CTYPE=German_Switzerland.1252    LC_MONETARY=German_Switzer
land.1252
## [4] LC_NUMERIC=C                        LC_TIME=German_Switzerland.1252
##
## attached base packages:
## [1] stats     graphics  grDevices utils     datasets  methods   base
##
## other attached packages:
## [1] visreg_2.4-1    lattice_0.20-34 nlme_3.1-131    reshape2_1.4.3  openxlsx_4.0.17
##
## loaded via a namespace (and not attached):
##  [1] Rcpp_0.12.16    digest_0.6.14   rprojroot_1.2   mime_0.5        grid_3.3.3      plyr_1.8.4      b
ackports_1.1.2
##  [8] magrittr_1.5    evaluate_0.10   highr_0.6       stringi_1.1.3   rmarkdown_1.4   tools_3.3.3     s
tringr_1.2.0
## [15] markdown_0.7.7  yaml_2.1.14     htmltools_0.3.5 knitr_1.15.1
```

References

R Core Team (2017). R: A language and environment for statistical computing. R Foundation for Statistical Computing, Vienna, Austria. URL https://www.R-project.org/.

Pinheiro J, Bates D, DebRoy S, Sarkar D and R Core Team (2017). *nlme: Linear and Nonlinear Mixed Effects Models*. R package version 3.1-131, <URL: https://CRAN.R-project.org/package=nlme>.

Patrick Breheny and Woodrow Burchett (2017). visreg: Visualization of Regression Models. R package version 2.4-1. https://CRAN.R-project.org/package=visreg
