# Supplementary figures and images for: Amide Proton Transfer Contrast Distribution in Different Brain Regions in Young Healthy Subjects
Source: Front Neurosci. 2019 May 22;13:520. doi: 10.3389/fnins.2019.00520 (PMC6538817; doi:10.3389/fnins.2019.00520)

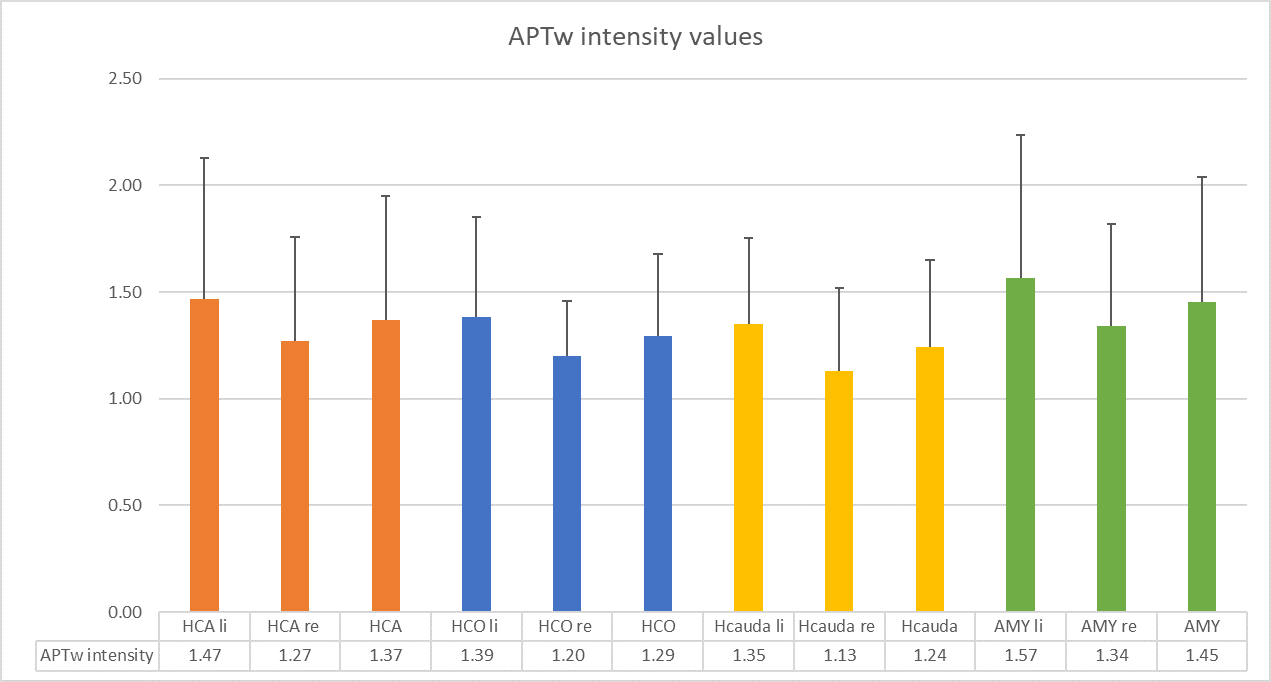

Supplement: Supplementary file 2 [file Image_1.TIFF]

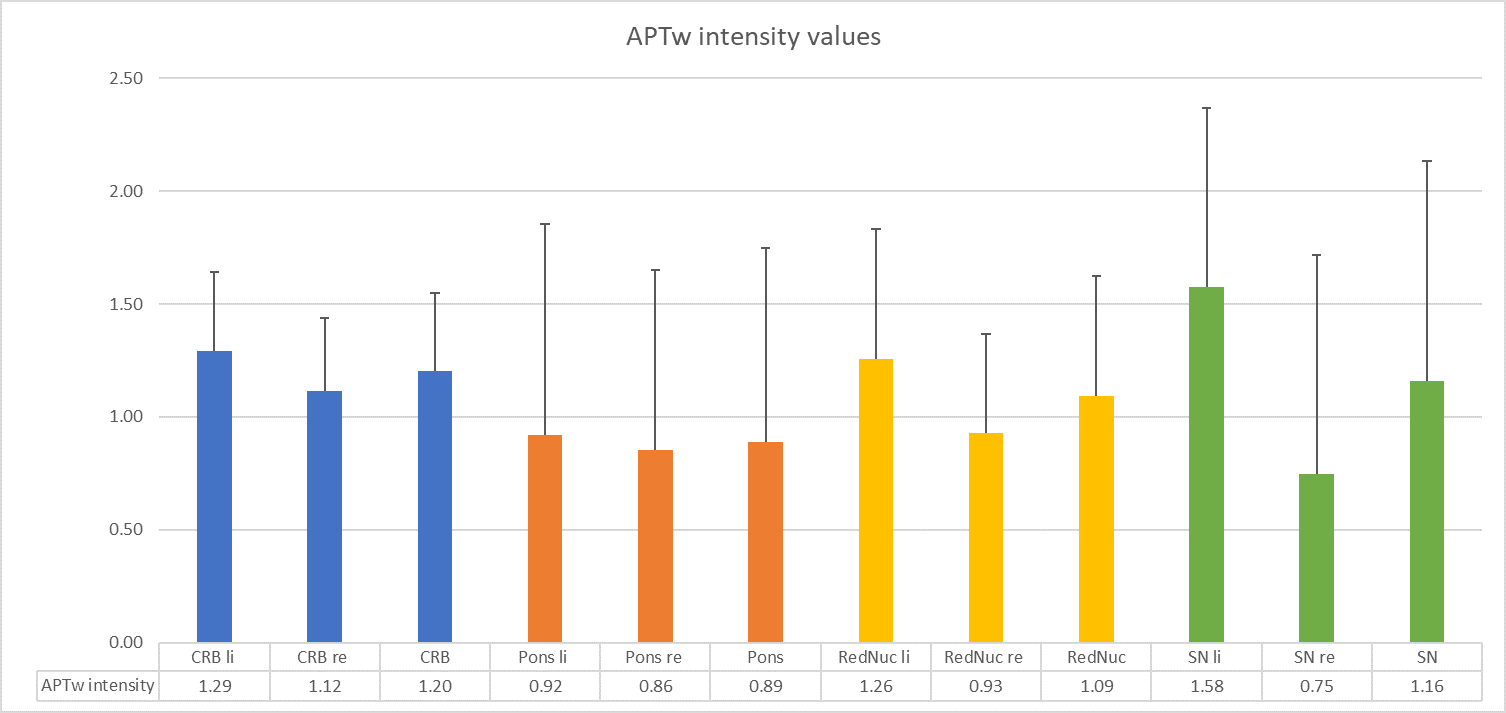

Supplement: Supplementary file 3 [file Image_2.TIFF]

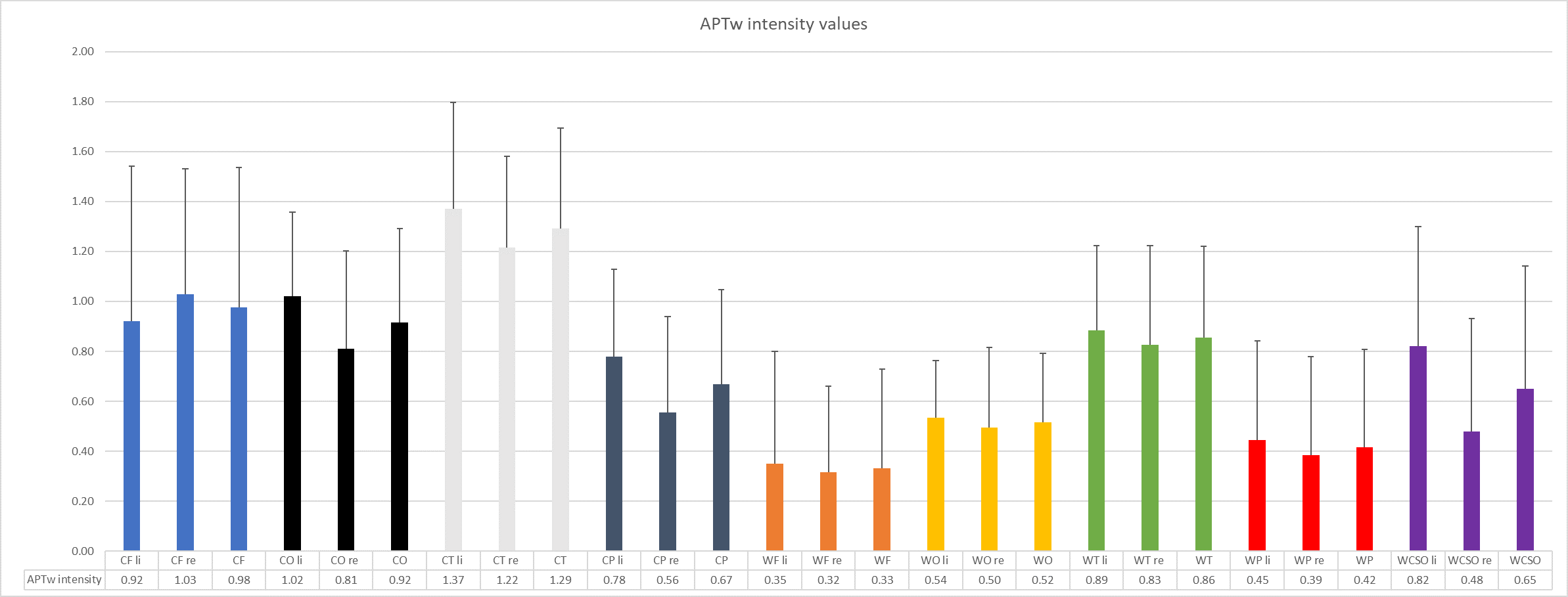

Supplement: Supplementary file 4 [file Image_3.TIFF]

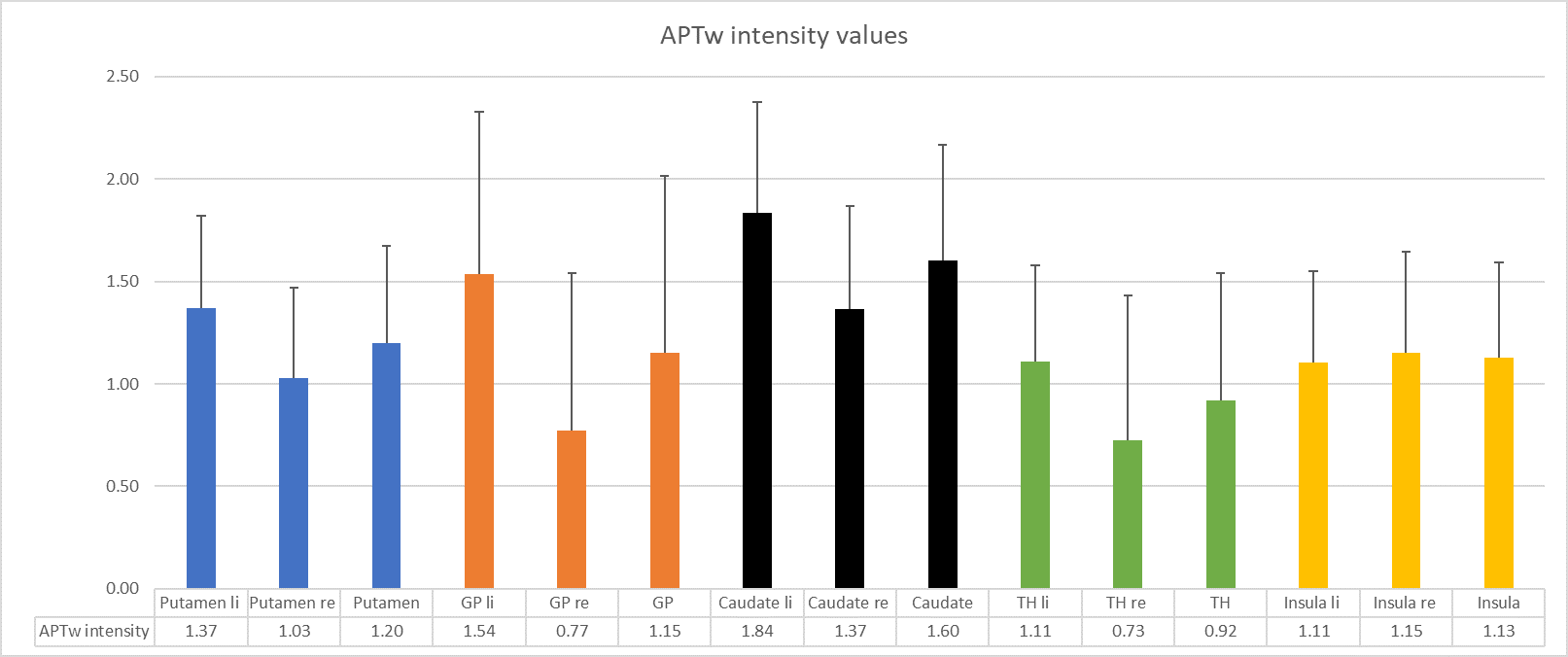

Supplement: Supplementary file 5 [file Image_4.TIFF]
