# Supplementary material for: Amide Proton Transfer Contrast Distribution in Different Brain Regions in Young Healthy Subjects
Source: Front Neurosci. 2019 May 22;13:520. doi: 10.3389/fnins.2019.00520 (PMC6538817; doi:10.3389/fnins.2019.00520)
Supplement: Supplementary file 6 [file Table_1.DOCX]

Supplementary Figures

Supplementary Fig.1 – Supplementary Fig. 4

Mean APTw intensity values (shown as coloured boxes) with standard deviations (upper whiskers) for left and right hemisphere and both hemispheres combined (bihemipsheric).

Supplementary Table 2

Data file containing the mean APTw signal intensity values for each volunteer and brain region.

Supplementary Table 3

Data file containing the minimum and maximum APTw signal intensity values for each volunteer and brain region.
